# American Resorts

**Published:** 1889-07

**Authors:** 


AMERICAN RESORTS, with notes upon their climate, by Bushrod W. James, A. M., M. D., Member of the American Association for the advancement of Science; The American Public Health Association; The Pennsylvania Historical Society; The Franklin Institute, and the academy of Natural Sciences, Philadelphia; the Society of Alaskan Natural History and Ethnology, Sitka, Alaska, etc., etc. With a translation from the German by Mr. S. Kauffman of those chapters of "Die Klimate der Erde," written by Dr. A. Woeikof, of St. Petersburg, Russia, that relate to North and South America and the Islands and Oceans contiguous thereto.

The above work contains 285 large oc. pages of exceedingly interesting reading matter, treated in 12 chapters as follows:

1, Medical Climatology. 2, Benefits and dangers of health resorts. 3, Seaside Resorts. 4, Fresh water resorts. 5, Mountain resorts. 5, Trips upon ocean, lake and river. 7, Mineral springs. 8, Summer resorts. 9, Winter resorts. 10, Therapeutics. 11, Mexico and South America. 13, Tropical ocean, etc.

The work contains a large and valuable map of the U. S., beautifully and we believe correctly executed.

